# Mapping the Oral–Nutrition–Brain Axis in Aging: A Scoping Review of Evidence Linking Oral Health, Nutrition, and Cognitive Outcomes

**DOI:** 10.3390/nu18213637

**Published:** 2026-10-31

**Authors:** Steffany Chamut, Holly Nowak, Saeideh Dadras, Rajni Kumari, Roberto Carlos Castrejón-Pérez, Jacqueline Pontes Monteiro, Kathaleen Briggs Early, Denisse Chamut, Melanie Morris, Joana Pico, Matt Glittenberg, HABS-HD Study Team, Marc Weisskopf

**Affiliations:** 1 School of Dental Medicine, Pacific Northwest University of Health Sciences, Yakima, WA 98901, USA; kearly@pnwu.edu; 2 Departments of Environmental Health and Epidemiology, Harvard T.H. Chan School of Public Health, Boston, MA 02115, USA; mweissko@hsph.harvard.edu; 3 The Health and Aging Brain Study-Health Disparities, Fort Worth, TX 76107, USA; rc.castrejon.perez@gmail.com (R.C.C.-P.); 4 School of Dental Medicine, University of Colorado Anschutz, Medical Campus, Aurora, CO 80045, USA; holly.nowak@cuanschutz.edu (H.N.); saeideh.dadras@cuanschutz.edu (S.D.); rajninemani@gmail.com (R.K.); 5 Familia Dental LLC, Midland, TX 79701, USA; 6 Wisconsin Alzheimer’s Disease Research Center, School of Medicine and Public Health, University of Wisconsin-Madison, Madison, WI 53706, USA; pontesmont@medicine.wisc.edu (J.P.M.); mpglitten@medicine.wisc.edu (M.G.); 7 College of Osteopathic Medicine, Pacific Northwest University of Health Sciences, Yakima, WA 98901, USA; 8 Next S-Miles, San Antonio, TX 78205, USA; nexts-miles@outlook.com; 9 School of Social Work, University of Washington, Seattle, WA 98195, USA; melmor@uw.edu; 10 Department of Nutrition, Dietetics, and Food Sciences Quinney, College of Agriculture and Natural Resources, Utah State University, Logan Campus, Logan, UT 84322, USA; joana.picocarbajo@usu.edu

**Keywords:** oral health, functional dentition, nutrition, Alzheimer’s disease and related dementias, periodontal disease, vitamins-B12-folate, homocysteine, healthy aging

## Abstract

**Background:** Oral health, nutrition, and neurocognitive health are increasingly recognized as components of healthy aging; however, their multidimensional relationships and plausible biological connections relevant to Alzheimer’s disease and related dementia (AD/ADRD) risk remain incompletely characterized. **Objective:** To systematically map evidence linking oral conditions and function, nutrition-related factors and biomarkers, and neurocognitive outcomes in adults aged ≥65 years, and conceptualize an integrative Oral–Nutrition–Brain Axis (ONBA). **Methods:** This scoping review followed Joanna Briggs Institute methodology and PRISMA-ScR guidance. Five electronic databases were searched for studies published from 2010 through June 2025. Evidence was harmonized across oral and functional indicators, nutritional/metabolic/inflammatory biomarkers, and neurological and cognitive outcomes. **Results:** Of 3415 records identified, 1988 unique records were screened, and 120 studies met eligibility criteria. Evidence was predominantly observational and geographically concentrated in East Asia. Periodontal inflammation, compromised dentition, impaired functional occlusion and masticatory performance were consistently associated with nutritional vulnerability, poorer cognitive performance, cognitive decline, and adverse AD/ADRD outcomes. Co-occurring nutritional and systemic markers included low body weight, hypoalbuminemia, low serum vitamin B12 and folate, hyperhomocysteinemia, and elevated inflammatory biomarkers. Mapped findings aligned across three proposed conceptual frameworks: inflammatory–microbial, nutritional–metabolic, and sensorimotor deafferentation axes. Studies incorporating neuroimaging biomarkers or integrating all three ONBA domains simultaneously remained limited. **Conclusions:** Oral health and functional dentition may represent key modifiable proxies associated with nutritional and neurocognitive vulnerability in older adults. Longitudinal studies incorporating harmonized oral, nutritional, inflammatory, and neurological phenotypes are needed to clarify temporal sequence, evaluate potential explanatory relationships, and inform multi-domain risk assessment for healthy cognitive aging.

## 1. Introduction

The World Health Organization (WHO) recognizes oral health as an integral component of overall health and well-being and describes healthy aging as maintaining the functional ability supporting well-being in older age. At the biological level, aging reflects the progressive accumulation of molecular and cellular changes that can reduce physical and cognitive capacity and increase disease vulnerability [1]. These trajectories are further influenced by nutritional vulnerability (susceptibility to inadequate nutritional intake or status due to biological, functional, or social factors) and malnutrition, compromising physical function, resilience, and cognitive health [1,2,3,4]. Importantly, these aging processes are neither linear nor uniform, varying substantially across individuals and diverse racial and ethnic population groups [1,5,6,7,8,9,10,11].

Oral diseases (ODs) and Alzheimer’s disease and related dementias (AD/ADRD) affect nearly 4 billion and 50 million people worldwide, respectively, posing profound public health, social and economic burdens [4,12,13,14,15,16,17,18]. Neuropathological changes associated with AD/ADRD can begin up to 30 years before dementia symptoms emerge, highlighting the critical need for early detection and modifiable risk identification [19,20,21]. Epidemiological evidence increasingly links oral-systemic health and cognitive trajectories [4,22,23,24,25]. On one hand, cognitive decline and natural tooth loss increase susceptibility to ODs due to diminished self-care abilities and neglected oral hygiene [19,26]. On the other hand, chronic oral pathologies are increasingly recognized as potentially modifiable risk factor driving the onset and progression to potentially modifiable factors associated with AD/ADRD vulnerability [27,28,29,30,31,32].

Despite this growing recognition, standard neurodegenerative frameworks rely heavily on traditional neurological markers, often omitting upstream oral, nutritional, and one-carbon metabolic indicators (e.g., vitamin B12, folate, homocysteine) [33]. While population-based studies link tooth loss and periodontitis with cognitive decline, limited evidence explains how oral conditions interact with indirect, low-cost peripheral markers (such as B-vitamin deficiencies, hyperhomocysteinemia, and systemic inflammatory mediators) to influence cognitive outcomes [33]. Impaired dentition and reduced masticatory efficiency directly restrict dietary quality and nutrient intake, making nutritional status a key potential mediator connecting oral breakdown to systemic metabolic and neurocognitive vulnerability [22,34]. The Health and Aging Brain Study–Health Disparities (HABS-HD) provides an important interdisciplinary framework for investigating cognitive aging across diverse populations through integrated clinical, biomarker, and health disparity characterization [9,11,15]. Motivated by this multidimensional approach, the present scoping review addresses a critical gap: the links connecting oral health conditions (e.g., orofacial pain, jaw dysfunction, functional dentition, chewing ability) and nutritional biomarkers to cognitive decline remain underexplored and unharmonized within a single conceptual framework [9,11,18,33].

To systematically map this evidence, we proposed an a priori conceptual framework (the Oral–Nutrition–Brain Axis [ONBA]) encompassing three hypothesized, interconnected biological pathways:

1. **The Inflammatory-Microbial Axis:** Suggests that chronic periodontal infections and polymicrobial dysbiosis elevate circulating pro-inflammatory cascades and neuroinflammatory responses [31,32].
2. **The Nutritional-Metabolic Axis:** Posits that structural oral alterations (tooth loss and impaired occlusal function) lead to dietary shifts toward soft, less nutrient-dense foods, potentially predisposing individuals to low body weight, reduced serum albumin, B-vitamin deficiencies, and elevated plasma homocysteine (a marker linked to microvascular injury and hippocampal vulnerability) [34,35].
3. **The Sensorimotor Deafferentation Axis:** Proposes that loss of functional dentition and occlusal contacts reduced trigeminal sensory feedback during mastication, which may attenuate regional cerebral blood flow to cognitive centers like the hippocampus [36,37,38].

By moving beyond traditional central neurological markers alone, this scoping review (ScR) aims to systematically map, synthesize, and identify evidence gaps regarding how oral health factors, nutritional biomarkers, and cognitive outcomes interact in older adults (≥65 years). By integrating evidence across these three intersecting domains into a unified ONBA framework, this review seeks to identify critical evidence gaps and generate a conceptual model to guide future longitudinal studies, harmonized risk modeling, and multi-domain preventive research in healthy cognitive aging [26,39,40].

## 2. Methods

### 2.1. Study Design and Methodological Framework

This scoping review (ScR) was conducted following the updated Joanna Briggs Institute (JBI) methodological guidance for scoping reviews [41,42]. Reporting was executed in strict compliance with the Preferred Reporting Items for Systematic Reviews and Meta-Analyses extension for Scoping Reviews (PRISMA-ScR) guidelines (Table S1) [41,42]. The review was conducted according to an *a priori* prospective protocol detailing the core objectives, research questions, Population, Concept, and Context (PCC) eligibility criteria, search strategy (Table S2), and data extraction plan, which was publicly registered on the Open Science Framework (OSF) repository (https://www.cos.io).

### 2.2. Inclusion Criteria (PCC Framework)

In alignment with JBI scoping review methodology, eligibility was structured around the Population, Concept, and Context (PCC) framework (Table 1):

**Population (P):** Older adults aged 65 years and older. Studies evaluating mixed-age cohorts were eligible only if data for participants aged ≥65 years were disaggregated or analyzed separately.

**Concept (C):** Research examining the multidimensional relationships across at least two of the three core domains defining the Oral–Nutritional–Brain axis (ONBA). Requiring multi-domain interaction was essential to capture across-domain interconnectedness rather than isolated single-domain outcomes:

1. **Oral Health, Functional Indicators, or Proxies (Block 1—Primary Exposures):** Quantitative and qualitative metrics of anatomical dentition status (natural teeth count, edentulism, DMFT index [Decay, Missing, Filling Theet]), functional masticatory performance (maximal bite force, chewing efficiency, functional masticatory units or functional occlusal pairs [FMUs]), prosthetic rehabilitation status (type, quality, and occlusal restoration of removable or fixed prostheses), and periodontal or local inflammatory burden (Probing Pocket Depth [PPD], Clinical Attachment Loss [CAL], Bleeding on Probing [BOP], Periodontal Inflamed Surface Area [PISA], oral dysbiosis/pathogens).
2. **Nutritional, Metabolic, and Inflammatory-Related Biomarkers (Block 2—Systemic Mediators):** Laboratory and metabolic intermediates acting as potential mediators or moderators, including B-complex vitamins (vitamin B12, folate), plasma homocysteine, serum albumin, CRP, pro-inflammatory cytokines (IL-6, TNF-α), or nutritional risk indices (Body Mass Index [BMI], Mini-Nutritional Assessment [MNA], Geriatric Nutritional Risk Index [GNRI]) [43,44].
3. **Neurological and Cognitive Outcomes (Block 3—Target Endpoints):** Objective indicators of cognitive vulnerability and AD/ADRD pathology, including standardized screening tools (MMSE [Minimal Mental State Examination], MoCA [Montreal Cognitive Assessment]), domain-specific neuropsychological assessments (memory, executive function, attention), clinical diagnoses of mild cognitive impairment (MCI), all-cause dementia, or AD/ADRD, and neuroimaging/pathological markers (grey matter volume, hippocampal atrophy, PET amyloid/tau deposition).

**Context (C):** Any epidemiological or clinical setting, encompassing community-dwelling populations, nursing homes/long term facilities, memory clinics, and population-based longitudinal studies or national administrative databases.

### 2.3. Exclusion Criteria

Studies were excluded if they failed to include participants aged ≥65 years or lacked subgroup reporting for this age demographic; evaluated only a single ONBA domain without examining interdomain links; were narrative reviews, commentary pieces, letters to the editor, editorials, or conference abstracts lacking full peer-reviewed text; utilized animal or in vitro model systems; or were published prior to 2010 or in languages other than English. Systematic reviews and meta-analyses were screened for reference tracking but excluded from primary mapping extraction to avoid duplicate participant representation.

### 2.4. Search Strategy and Information Sources

A comprehensive systematic literature search was developed and executed by an expert health sciences librarian on 17 June 2025. The search was performed across five major electronic databases: MEDLINE ALL (Ovid); Embase (Embase.com); Web of Science Core Collection; Cochrane Database of Systematic Reviews (Cochrane Library, Wiley); Cochrane Central Register of Controlled Trials (Cochrane Library, Wiley). The search strategy covered studies published between 1 January 2010 and 30 June 2025 (capturing all indexed pre-prints and ahead-of-print records through the execution month). Language was restricted to English, which was recognized *a priori* as a potential language bias limitation.

Following PRESS (Peer Review of Electronic Search Strategies) principles, search strings combined Controlled Vocabulary (MeSH in MEDLINE, Emtree in Embase) and free-text keywords covering three primary blocks:

- **Block 1:** “oral health”, “tooth loss”, “functional dentition”, “masticatory performance”, “occlusal force”, “chewing efficiency”, “periodontitis”, “periodontal disease”, “oral frailty”, and “orofacial pain”.
- **Block 2:** “vitamin B12”, “folate”, “homocysteine”, “albumin”, “C-RP”, “interleukin-6”, “TNF-α”, “malnutrition”, and “nutritional status”.
- **Block 3:** “dementia”, “Alzheimer disease”, “mild cognitive impairment”, “cognitive decline”, “MMSE”, “MoCA”, “neuroimaging”, and “brain atrophy”.

The full search strategies for each database are available in Table S2. Reference list screening of key reviews and forward citation tracking of included studies were additionally performed to ensure literature saturation.

### 2.5. Evidence Screening and Selection

All retrieved citations were exported to EndNote (Version 2025) for reference management and automated duplication removal. De-duplicated records were uploaded to Covidence for independent screening. Pilot testing was conducted on 20 citations to establish high inter-reviewer agreement (Cohen’s k > 0.85). Study selection followed an iterative, two-stage process:

- **Title and Abstract Screening:** Two independent reviewers (HN, SD) screened all titles and abstracts against the PCC criteria.
- **Full-Text Evaluation:** Potentially eligible studies underwent full-text retrieval and independent evaluation by the same two reviewers.

**Figure 1:**
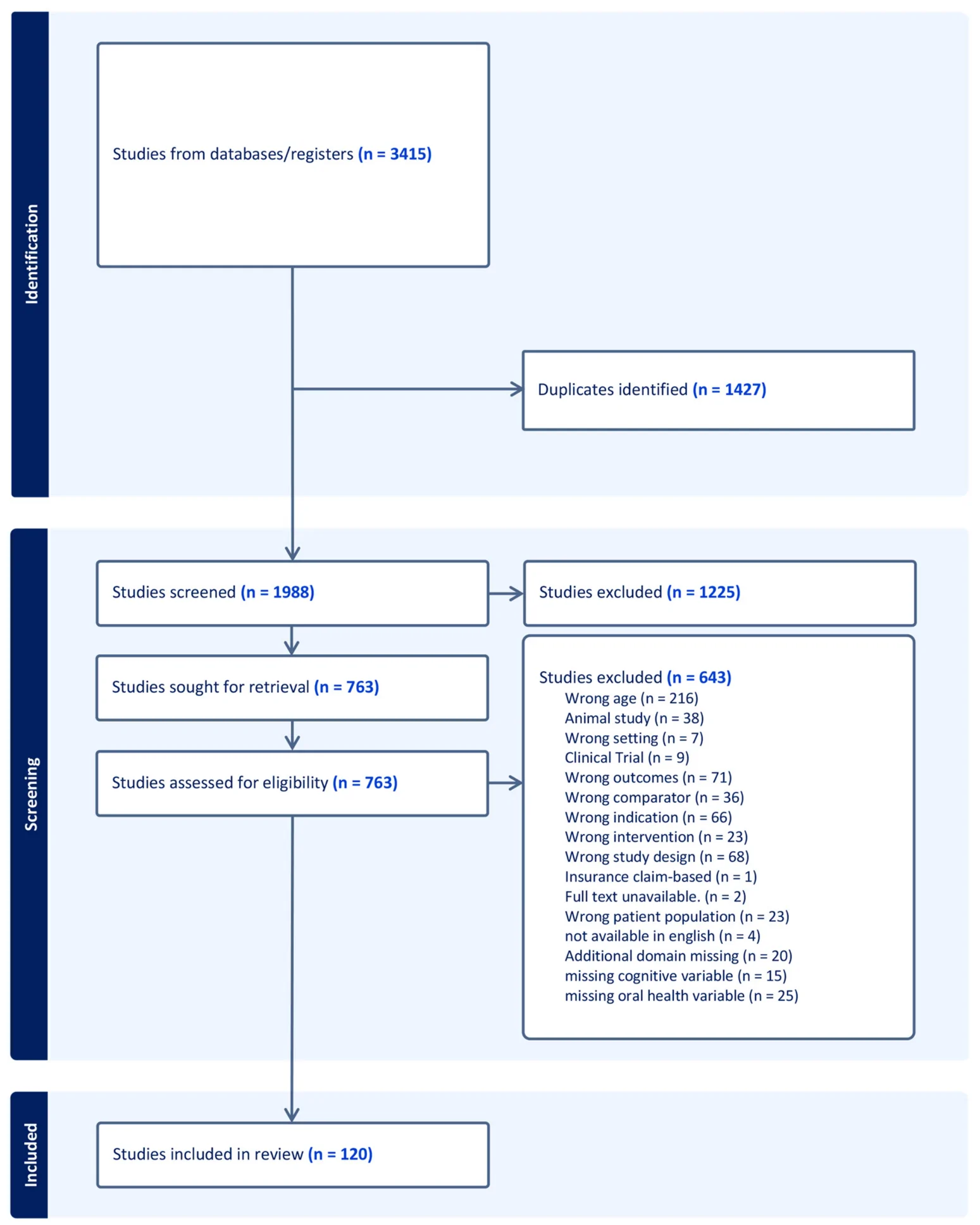
PRISMA-ScR flow diagram.

Disagreements at either stage were resolved via consensus or arbitration by senior reviewers (RK, SC). Excluded full-text studies were documented with specific reasons (Figure 1). In accordance with updated JBI guidance and PRISMA-ScR standards, formal critical appraisal or risk of bias scoring was not performed, as the primary objective of this scoping review was to map the evidence landscape and gaps rather than evaluate intervention efficacy or generate a single pooled effect size [41,42].

### 2.6. Data Extraction (Charting) and Harmonization

Data extraction was structured using a pre-developed, standardized data charting form created in Microsoft Excel. The standardized charting form was developed to maintain data quality through double-checking and independent validation, ensuring accuracy and completeness (Table 1). The extraction form was piloted by the review team on three representative studies prior to implementation. Data extraction was performed in duplicate (HN, SD) to minimize extraction errors, with consensus verification by a third reviewer (SC). For each included study (n = 120), six primary parameters were charted:

- **Study Metadata:** Lead author, publication year, title, journal, country of origin, and specific cohort identifier (e.g., SONIC, Fujiwara-kyo, Hiyasama, CLHLS, HABS-HD) [9,11,15,17,45,46,47,48].
- **Study Design and Methodology:** Sampling methodology, study design (cross-sectional, longitudinal cohort, case–control, systematic review, trial), sample size, follow-up duration, and statistical modeling approach.
- **Participants Characteristics:** Age criteria, mean age, setting (community-dwelling, nursing home/long-term care, memory clinic), and cognitive baseline status.
- **Oral Health Indicators (Harmonized):** Quantitative natural tooth count, edentulism, DMFT index, masticatory performance (bite force, chewing efficiency, FMUs), prosthetic status (denture quality and wear), and periodontal metrics (PPD, CAL, BOP, PISA, bacterial loads).
- **Nutritional and Inflammatory Biomarkers:** Biochemical assays (B12, folate, homocysteine, CRP, IL-6, TNF-α, albumin) and anthropometric indices (BMI, MNA).
- **Cognitive and Neurological Outcomes:** Screening scores (MMSE, MoCA), clinical diagnostic categories (MCI, AD, ADRD), domain-specific cognitive metrics, and neuroimaging markers.

### 2.7. Data Analysis

In accordance with Peters et al. [41,42], extracted data were analyzed using descriptive statistics (frequency counts, percentages, and geographical distributions) and qualitative conceptual synthesis. Data synthesis was organized around the three predefined biological domains of the ONBA. Findings are visually presented through structured mapping tables, geographic breakdowns, and a conceptual framework diagram illustrating potential associations, candidate mediating pathways, and evidence gaps in linking oral health and nutritional biomarkers to AD/ADRD risk.

## 3. Results

The initial search database yielded 3415 records across MEDLINE, Embase, Web of Science, and the Cochrane databases. Following automated and manual de-duplication, 1988 unique records underwent title and abstract screening. Full-text evaluation of eligible manuscripts identified 120 studies published between 2010 and June 2025 that met all PCC eligibility criteria for inclusion in this ScR (Table 2; see PRISMA Figure 1).

### 3.1. Methodological and Geographical Landscape

Research examining oral health, nutrition, and cognitive aging has increased globally, with a distinct concentration of observational studies across East Asia and Western nations. The included literature was predominantly observational (94.2%), comprising 52% cross-sectional studies and 38.3% prospective or retrospective cohort studies [17,19,27,30,37,45,46,47,48,49,50,51,52,53,54,55,56,57,58,59,60,61,62,63,64,65,66,67,68,69,70,71,72,73,74]. Case–control studies represented 4.2% [39,75,76,77,78], systematic reviews/meta-analyses represented 3.3% [25,79,80,81,82,83,84,85,86,87], and original interventional trials/prosthetic rehabilitation studies represented 1.7% [88,89,90,91].

Japan contributed the largest body of evidence (35.8%) [14,17,23,36,38,48,49,50,51,52,54,56,57,61,62,63,65,68,73,75,89,92,93,94,95,96,97,98,99,100,101,102,103,104,105,106,107,108,109,110,111,112,113,114,115], followed by the US (9.2%) [15,30,60,66,116,117,118,119], China (7.5%) [26,46,53,67,74,82,105,120,121,122], South Korea (6.7%) [36,58,108,123,124,125,126,127,128], Taiwan (3.3%) [27,40,59,129], and Finland (3.3%) [81,130,131,132,133]. Additional evidence originated from Brazil, Italy, the Netherlands, Australia, India, Sweden, Singapore, and Turkey [45,69,76,81,88,91,130,133,134,135,136,137,138,139,140,141,142,143].

Studies predominantly evaluated community-dwelling older adults (81.7%) [14,17,27,37,45,46,48,49,50,51,52,54,55,56,61,62,64,65,68,71,73,74,75,99,103,104,138,140], with additional subset from long-term care and nursing home populations (11.7%) and memory-clinic outpatients (6.6%) [78,91,97,110,117,120,130,132,135,137,141,142,144,145,146,147,148,149,150,151]. Cognitive vulnerability was primarily assessed using global screening tools (MMSE, MoCA; 55.8%) [45,134,135,144,152], clinical diagnostic classification (MCI, AD, ADRD; 71.7%) [19,23,26,40,47,59,63,76,77,81,88,110,131,134,141,146], domain-specific neuropsychological batteries (26.7%), and neuroimaging/fluid biomarkers (10.8%) [14,32,90,96,98] (Table 2).

### 3.2. Evidence Across the Oral–Nutrition–Brain Axis (ONBA)

#### 3.2.1. Block 1: Oral Health Exposures, Structural Integrity, and Functional Capacity

The mapped literature evaluated oral health across four harmonized functional and pathological categories (dentition status, functional and masticatory performance, prosthetic rehabilitation, and periodontal and inflammatory status), consistently reporting that adverse oral health metrics are associated with cognitive impairment.

1. **Dentition Status:** Natural tooth count was the most frequently investigated oral exposure. Across prospective cohorts, maintaining ≥20 natural teeth was consistently associated with a reduced risk of cognitive decline [17,19,50,53,54,55,56,57,72,73,129]. Conversely, tooth loss (<10 teeth) and complete edentulism exhibited a dose–response relationship with accelerated cognitive decline and incident dementia [17,19,46,47,67,72,74,145]. In institutionalized and nursing home settings, complete edentulism correlated with clinical dementia severity [39,57,110,119,127,145,146,149,151]. Studies employing a Decayed, Missing, Filled Teeth (DMFT) index further confirmed that cumulative, untreated dental caries burden differentiates cognitively impaired from cognitively intact cohorts [120,122,139].
2. **Functional and Masticatory Performance:** Beyond static tooth count, objective measures of masticatory physiology revealed notable associations with cognitive performance. Reduced maximal occlusal force, fewer FMUs, and impaired chewing efficiency (objectively measured via two-color wax or color-changing gum mixing tests) were independently associated with lower MMSE/MoCA scores and heightened MCI prevalence [36,48,49,64,93,108,111,113,126,127,137,142,144,153]. Patients with diagnosed AD exhibited prolonged masticatory cycle times, reduced chewing velocity, and impaired grinding capacity compared to age-matched controls [88,97,134,137,153].
3. **Prosthetic Rehabilitation:** Prosthetic status functioned as a key modifiable buffer. Across the longitudinal cohort, complete or partial denture wear was associated with attenuated cognitive risk related to tooth loss [63,67,78,88,129,152]. Effective prosthetic restoration improved chewing performance and bite force, which in turn correlated with stabilized cognitive trajectories and preserved regional gray matter volume on magnetic resonance imaging (MRI) [14,88,90]. However, the quality and functional fit of the prosthesis were critical: ill-fitting dentures or un-rehabilitated edentulism yielded cognitive trajectories comparable to severe untreated tooth loss [152].
4. Periodontal and Local Inflammatory Status. While tooth loss reflects cumulative lifetime disease, measures of active periodontitis (including PPD, CAL, BOP, and PISA) capture ongoing biological exposure. High periodontal inflammatory burden and elevated systemic/salivary loads of periodontal pathogens (*Porphyromonas gingivalis*, *Fusobacterium nucleatum*) were significantly associated with lower cognitive scores, faster 6-month cognitive decline, and increased incident MCI/AD [81] (Table 2).

#### 3.2.2. Block 2: Nutrition-Related Biomarkers and Inflammatory Intermediates

A major subset of included studies (40.8%) evaluated laboratory, somatic, and metabolic biomarkers alongside oral health, identifying potential biological intermediates linking oral breakdown to neurocognitive vulnerability [27,45,48,49,50,52,53,61,63,65,68,69,71,73,75,76,94,95,97,103,104,105,109,111,117,123,129,134,137,139,141,142,145,148,150,151,152,153,154,155,156].

1. **Nutritional and Somatic Vulnerability:** Impaired mastication and severe tooth loss were consistently linked to markers of undernutrition and nutritional vulnerability (low BMI, unintentional weight loss, reduced nutritional status, and lower serum albumin) [53,54,56,76,87,88,110]. Longitudinal path analyses supported nutritional status and reduced dietary intake (e.g., fruit, vegetable, and fibrous avoidance) as potential partial mediators linking tooth loss and incident cognitive decline [54].
2. **Micronutrient and One-Carbon Metabolism (B12, Folate, Homocysteine):** Studies evaluating micronutrient-related pathways reported that masticatory dysfunction alters dietary patterns, compromising the intake and absorption of essential B-complex vitamins [61,65,69,76,93,108]. Deficiencies in serum B12 and folate were frequently co-observed with elevated plasma homocysteine concentrations [69,76]. Elevated homocysteine, in turn, correlated directly with executive dysfunction, memory impairment, and microvascular white matter burden, providing additional evidence for potential metabolic pathways contributing to cognitive vulnerability [65,69,157] (Table 3).
3. Systemic Inflammatory Cascades: Active periodontal disease and high PISA scores were significantly correlated with elevated circulating inflammatory biomarkers, specifically CRP, IL-6, and Interleukin-1beta [23,30,52,59,133,155]. Studies incorporating multivariable path modeling suggested that systemic inflammation may act as a candidate mediator linking local oral infection and cognitive decline [52,133,155] (Table 3).

#### 3.2.3. Exploratory Synthesis of the Oral–Nutritional–Brain Axis (ONBA)

Neurological and cognitive outcomes represented the brain-health component of the ONBA literature ranged from global cognitive screening and preclinical memory impairment to clinically diagnosed MCI, dementia, and AD [39,55,72,74,108,119,120,121,123,132,136,139,141,145,146,150]. MMSE and MoCA were the most frequently used cognitive measures, with poorer dentition, masticatory dysfunction, and periodontal disease generally associated with lower cognitive performance or greater cognitive decline [26,35,36,45,48,52,68,95,99,104,111,113,119,152,153,155]. Additional studies identified associations with specific memory deficits and interactions with APOE ε4 susceptibility [75,77,158]. A smaller body of neuroimaging evidence extended these findings to structural and pathological neurological markers, linking adverse oral health with differences in brain volume and AD-vulnerable regions on MRI, as well as greater tau deposition on PET [14,32,90,96] (Table 3).

**Figure 2:**
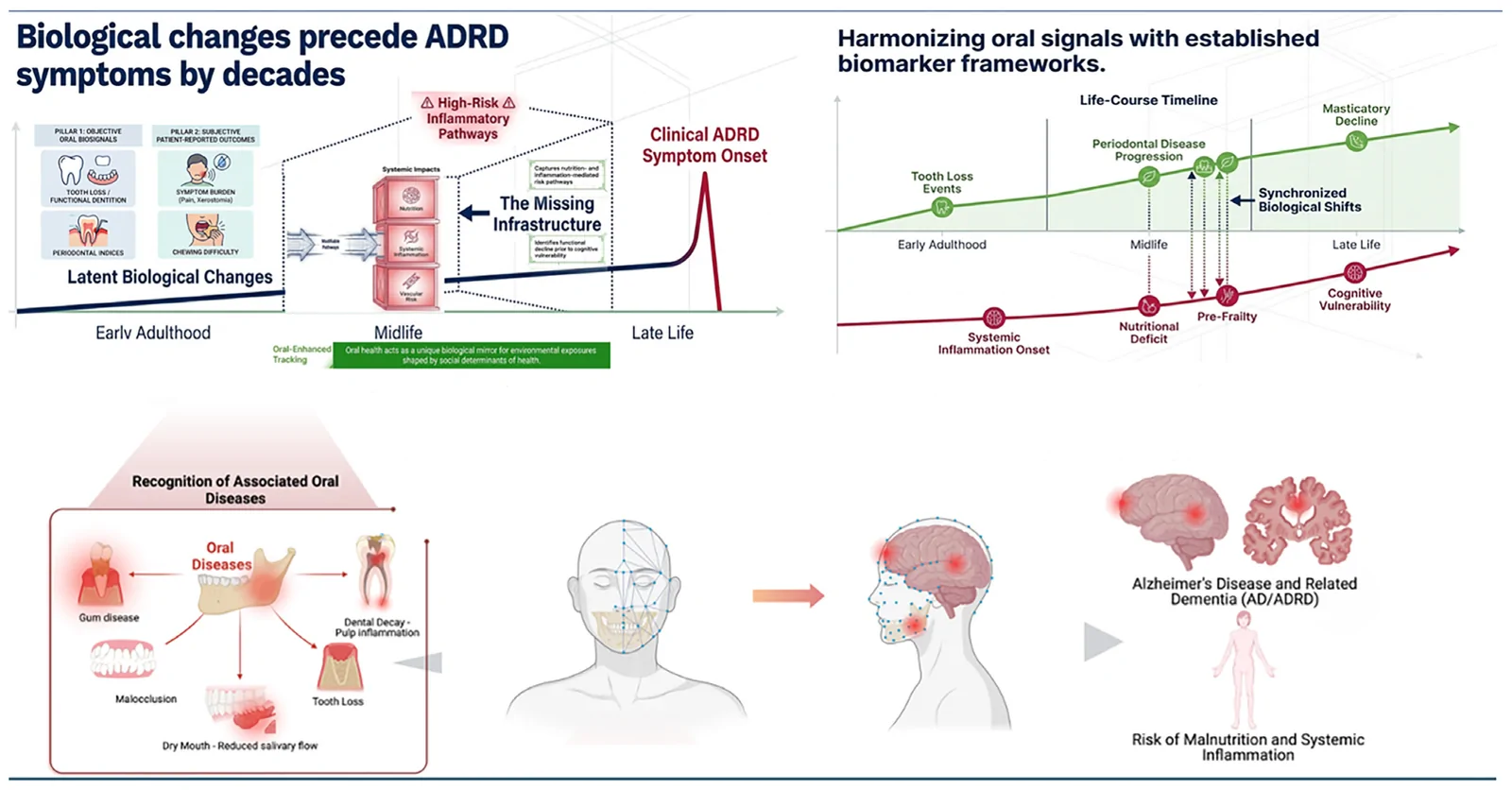
Conceptual life-course framework illustrating how oral disease, tooth loss, periodontal inflammation, and masticatory decline may converge with nutritional, inflammatory, vascular, and systemic pathways preceding cognitive vulnerability and clinical AD/ADRD.

When synthesized across the 120-study matrix, the literature provides a conceptual framework mapping three hypothesized biological pathways linking oral health to AD/ADRD risk:

1. **The Inflammatory-Microbial Axis:** Suggests that chronic periodontitis maintains persistent low-grade systemic inflammatory signaling while permitting oral pathogens (*P. gingivalis*) and virulence factors (gingipains) to cross the BBB, potentially driving microglial priming, neuroinflammation, and amyloid/tau deposition [25,30,81,155] (Table 3).
2. **The Nutritional-Metabolic Axis:** Posits that functional tooth loss is associated with dietary shifts toward soft, nutrient-depleted foods, contributing to B-vitamin (B12/Folate) insufficiency and impairing one-carbon methylation. The resulting hyperhomocysteinemia is hypothesized to exacerbate vascular endothelial dysfunction, microvascular brain injury, and hippocampal vulnerability [61,69,75,76,93,141,145,150] (Table 3).
3. **The Sensorimotor Deafferentation Axis:** Proposes that loss of posterior occlusal contacts and periodontal mechanoreceptors deprives the central nervous system of vital trigeminal sensory feedback during mastication. Neuroimaging studies suggest that reduced masticatory stimulation correlates with decreased regional cerebral blood flow to the hippocampus and prefrontal cortex, representing a candidate pathway for accelerated structural brain atrophy independent of inflammatory or nutritional factors [14,36,48,49,63,85,90,96,112,113] (Table 3). These interconnected life-course pathways and their conceptual integration with established AD/ADRD biomarker frameworks are illustrated in Figure 2.

#### 3.2.4. Key Exploratory Takeaways for Risk Modeling and Harmonization

Synthesis of the mapped literature indicates that tooth loss serves as a static, cumulative biomarker of historical biological aging and structural oral health disparities [14,71,133,145,154,159]; whereas periodontitis represents an active, ongoing biological exposure driving systemic inflammation [23,27,30,52,81,133,155]. The co-occurrence of severe tooth loss, periodontitis, B-vitamin deficiency, and hyperhomocysteinemia forms a high-risk oral-vascular-inflammatory cluster associated with accelerated cognitive decline and AD/ADRD vulnerability.

However, several critical evidence gaps emerged across the mapped landscape:

- Geographic Concentration: Over 65% of included studies originated from East Asia (predominantly Japan, China, South Korea, and Taiwan), limiting the direct generalizability of specific masticatory and dietary thresholds to Western populations or diverse health disparity cohorts.
- Scarcity of Multi-Domain Integration: Less than 12% of included studies evaluated all three ONBA domains (oral, nutritional, and neurocognitive) simultaneously within a single analytical framework.
- Limited Biomarker and Neuroimaging Coverage: Only 10.8% of studies incorporated objective neuroimaging (MRI volume, white matter hyperintensities) of fluid/PET biomarkers (*Aβ*, tau), with most relying on global cognitive screening tools (MMSE, MoCA).
- Rarity of Interventional Trials: Only 2 of the 120 studies (1.7%) evaluated clinical interventions (e.g., prosthetic restoration or combined dietary advice), highlighting a critical lack of interventional evidence to determine whether restoring oral function influences cognitive trajectories.

## 4. Discussion

This ScR mapped 120 studies published between 2010 and 2025, systematically mapping the multidimensional relationship across oral health, nutrition-related biomarkers, and cognitive outcomes in older adults (≥65 years). By operationalizing a structured PCC framework, our synthesis indicates that poor oral health (severe tooth loss, loss of functional occlusion, impaired mastication, and active periodontitis) is consistently associated with cognitive vulnerability, accelerated cognitive decline, and increased risk of AD/ADRD.

Rather than viewing dental conditions in isolation, the mapped literature supports an integrative Oral-Nutritional-Neurodegenerative framework operating across three distinct biological pathways:

- **The Inflammatory-Microbial Axis:** Suggests that active periodontitis maintains persistent systemic inflammatory signaling (elevated CRP, IL-6, TNF-*α*) and permits periopathogens (*Porphyromonas gingivalis*) and their virulence factors to compromise BBB integrity, potentially fueling microglial priming and neuroinflammation [27,30,32,52,81,133,155,160].
- **The Nutritional-Metabolic Axis:** Posits that reduced functional dentition and occlusal loss are associated with dietary shifts toward soft, processed foods, predisposing individuals to subclinical malnutrition, low serum albumin, and B-vitamin (B12 and folate) depletion [61,69,75,93,150]. This metabolic impairment elevates circulating plasma homocysteine, an established marker linked to vascular brain injury and hippocampal vulnerability [61,69,75,93,150].
- **The Sensorimotor Deafferentation Axis:** Proposes that loss of posterior occlusal contacts and periodontal mechanoreceptors deprives the central nervous system of vital trigeminal sensory feedback during mastication, which is hypothesized to correlate with reduced regional cerebral blood flow and structural volume changes in memory centers [14,36,49,63,85,90,96,98,112,113].

### 4.1. Methodological Synthesis Across Epidemiological Designs

A key objective of this ScR was to evaluate how evidence aligns across different study designs. While marked variations in sample sizes and regional populations exist, converging patterns emerged across epidemiological methodologies, highlighting consistent associations while exposing design-specific analytical limits.

**Longitudinal studies** provided the strongest evidence regarding temporal sequences linking poor oral health and neurocognitive decline [19,27,40,45,46,47,48,49,50,51,52,53,54,55,56,57,58,59,129]. Well-characterized cohorts (the Hisayama Study, AGES, and Ohasama Study in Japan, CLHLS in China, ELSA in the UK, and Health ABC in the US) consistently reported a dose–response relationship between tooth loss and incident dementia or cognitive decline [17,53,54,55,56,61,62,73]. Cohorts evaluating functional parameters confirmed that reduced bite force and objective chewing disability at baseline independently predict 3- to 10-year cognitive decline [67]. Furthermore, longitudinal path analyses supported dietary quality, body weight, and nutrient intake as potential partial mediators linking baseline tooth loss to subsequent cognitive trajectories [48,53,54,61,64,67,70,73,93].

**Cross-sectional studies** across community-dwelling individuals, nursing home residents, and clinical outpatients showed high concordance regarding functional capacity and cognitive impairment severity [13,14,32,35,36,81,93,103,104,108,113,115,122,123,124,126,128,130,131,132,133,134,136,137,139,140,141,142,145,159]. Across diverse settings, individuals with MCI and diagnosed AD displayed significantly lower natural tooth counts, reduced FMUs, weaker bite force, and prolonged chewing cycles compared to cognitively intact controls [88,97,103,105,112,120,122,126,134,135,136,137,139,142,144,153,158]. Cross-sectional biomarker studies consistently identified significant correlations between active periodontal disease (elevated PISA/PDD) and systemic inflammatory markers (CRP, IL-6), as well as between masticatory dysfunction and B-vitamin/homocysteine dysregulation [23,27,32,76,81,93,124,133,151,155]. **Case–control studies** differentiated clinical AD cohorts from non-demented controls based on oral disease burden and periodontal microbial profiles (e.g., *P. Gingivalis* antibody titers) [39,40,78,81,85,105,129].

**Interventional trials and prosthetic rehabilitation studies** provided preliminary observational insights: restoring functional occlusion via complete or partial dentures improved maximal bite force, increased chewing efficiency, and was associated with altered regional cerebral blood flow or cortical activity on neuroimaging [85,88,90,142,146]. However, these studies noted that while prosthetic rehabilitation attenuates cognitive decline risk, functional masticatory performance in rehabilitated edentulous older people rarely reaches the baseline performance of natural, fully dentate individuals [27,40,67,88,110,129,134,146,152].

### 4.2. Exploratory Risk Clustering and Biological Synergies

Synthesis of the 120 included studies indicates that tooth loss and active periodontitis represent distinct clinical exposures within neurodegenerative risk models. When periodontitis and elevated homocysteine co-occur, they form a high-risk oral–vascular–inflammatory risk cluster [30,32,69,75,76,133,155]. Inflamed periodontal tissues disrupt chewing efficiency, predisposing individuals to B-vitamin (B12/Folate) deficiency and impaired one-carbon methylation [61,69,75,76,93,141,145,153]. The resulting hyperhomocysteinemia is hypothesized to induce endovascular injury, while circulating cytokines (TNF-*α*, IL-6) exacerbate BBB permeability [30,32,69,76,81,103,133,155]. Concurrently, reduced masticatory movement decreases trigeminal afferent input to the brainstem and hippocampus, potentially attenuating regional perfusion [14,36,49,63,85,90,96].

### 4.3. Translational Hypotheses and Policy Context

While the mapped evidence remains predominantly observational, these findings support potential translational hypotheses for geriatric clinical frameworks, encouraging a transition from siloed dental and medical care toward integrated oral–nutritional–cognitive models. Comprehensive geriatric research protocols should evaluate whether incorporating low-cost oral functional metrics (e.g., self-reported chewing difficulty, simple bite force assays, or FMU counts) alongside nutritional panels (serum albumin, B12, folate, homocysteine) provides accessible risk proxies for early cognitive vulnerability screening. Preserving natural functional dentition (≥20 teeth) and maintaining periodontal therapy represent key target areas for mid-to-late-life preventive strategies [14,36,49,63,85,90,96]. For edentulous or partially dentate older adults, future studies should evaluate whether timely prosthetic rehabilitation combined with targeted B-vitamin supplementation can mitigate nutritional and sensorimotor drivers of cognitive decline.

International healthcare structures demonstrate marked contrasts in late-life oral healthcare provision.

Japan, for instance, combines high life expectancy with universal health coverage and longstanding national initiatives, such as the “8020 Campaign” (promoting retention of at least 20 natural teeth at age 80) [102]. In contrast, access to dental care among older adults in the United States remains fragmented, as traditional Medicare excludes routine preventive and restorative dental care, creating significant financial barriers that disproportionately affect socioeconomically disadvantaged and health disparity populations (such as those evaluated in HABS-HD) [116,161,162,163,164,165,166].

### 4.4. Methodological Limitations

Several methodological limitations inherent to scoping reviews and the underlying literature base must be acknowledged:

1. Language Restriction: Restricting eligibility to English-language publications may have introduced language bias, particularly given that over half of the included evidence originated from West Asia.
2. Observational Predominance: The heavy reliance on cross-sectional (52.5%) and observational cohort (38.3%) studies restricts causal inference and temporal interpretation.
3. Absence of Critical Appraisal: In accordance with JBI scoping review standards, formal risk of bias scoring was not performed, preventing the distinction between stronger and weaker evidence within mapped associations.
4. Non-Unique Sample Tallying: Cumulative sample sizes across included studies should not be interpreted as unique individual participants, as large longitudinal cohorts (e.g., Hiyasame, CLHLS, Fujiwara-kyo) contributed multiple publications across different follow-up waves.
5. Exposure and Outcome Heterogeneity: Marked heterogeneity in definitions of tooth loss, periodontal disease (ranging from single-item self-reports to full-mouth PISA examinations), masticatory performance, and cognitive metrics (ranging from MMSE screening to neuroimaging/PET markers) limits direct comparability or meta-analytic pooling.
6. A Priori Framework Categorization: The three-axis ONBA framework was defined *a priori* and may have influenced the categorization and qualitative synthesis of evidence.
7. Domain Multiplicity Threshold: The requirement for studies to evaluate at least two ONBA domains may have excluded relevant single-domain studies while restricting the ability to quantify fully integrated three-domain evidence.
8. Conceptual Mediation vs. Causal Testing: Several proposed mediating pathways were inferred conceptually from cross-sectional correlations rather than directly confirmed using formal causal mediation models or longitudinal path analyses. Reverse causation (e.g., early cognitive decline leading to oral hygiene neglect) and residual confounding (from socioeconomic status, lifelong dental access, and health literacy) remain possible.

### 4.5. Future Perspectives

Future research should move beyond isolated oral, nutritional, inflammatory, and cognitive measurements toward longitudinal, multi-domain studies capable of evaluating temporal and bidirectional relationships. Future cohorts should harmonize standardized measures of dentition, periodontal inflammation (PISA), masticatory performance (FMUs, bite force), dietary intake, nutritional status, one-carbon biomarkers (B12, folate, homocysteine), inflammatory cytokines, global cognition, and neuroimaging. Analytical models must incorporate frailty, socioeconomic status, social isolation, depression, healthcare access, and caregiver support as potential confounders and effect modifiers.

Furthermore, research should leverage multi-ethnic cohorts (such as HABS-HD) to validate whether ONBA associations persist across diverse racial and ethnic groups. Advanced causal inference methods, including Mendelian randomization and structural equation modeling, should be applied to disentangle directional pathways. Finally, well-designed randomized controlled trials are needed to determine whether combined periodontal care, high-quality prosthetic rehabilitation, and targeted nutritional supplementation can favorably modify cognitive trajectories and mitigate AD/ADRD risk in vulnerable older populations.

## 5. Conclusions

This scoping review indicates consistent associations connecting oral diseases, loss of functional dentition, and masticatory breakdown to neurocognitive vulnerability and AD/ADRD risk. Rather than establishing direct causality, the mapped literature supports a plausible conceptual framework operating across three interconnected pathways: the inflammatory-microbial, nutritional-metabolic, and sensorimotor deafferentation axes. While these biological mechanisms represent plausible conduits linking oral breakdown to neurodegeneration, their relative individual contributions and exact temporal directionality remain to be fully clarified. These conclusions must be interpreted in light of key evidence gaps identified across the mapped landscape, including the heavy predominance of observational and cross-sectional study designs (94.2%), significant geographical concentration in West Asia (>56%), marked heterogeneity in oral and cognitive outcome definitions, and a scarcity of studies evaluating all three ONBA domains simultaneously. Ultimately, determining whether preserving oral health, adequate nutritional status, and masticatory capacity contributes to healthy cognitive aging represents a critical interdisciplinary priority for preserving functional independence and quality of life in older adults.

## Figures and Tables

**Table 1:** Search strategy key concepts, ONBA domain definitions, and eligibility criteria based on the Population, Concept, and Context (PCC) scoping review framework (*N* = 120).

Framework Element	ONBA Domain/Block	Operational Focus & Subcategories	Methodological Role & Eligibility Criteria
Population (P)	Target Age Demographic	Community-dwelling older adults; long-term care / nursing home populations; memory clinic outpatients; healthy aging cohorts; oldest-old cohorts (≥85 years).	Inclusion Rule: Adults aged ≥65 years. Mixed-age cohorts were eligible only if data for participants aged ≥65 years were disaggregated or analyzed separately.
Concept (C)	Domain 1 (Block 1): Oral Health Indicators, Functional Capacity & Proxies	• Anatomical Dentition: Natural tooth count, complete edentulism, DMFT index.	Primary Exposures & Proxies: Evaluated across structural, functional, prosthetic, and inflammatory oral health dimensions.
		• Masticatory Physiology: Functional Masticatory Units (FMUs), maximal bite/occlusal force, chewing efficiency (mixing tests).	
		• Prosthetic Status: Complete/partial denture wear, functional fit/quality.	
		• Periodontal Burden: Probing Pocket Depth (PPD), Clinical Attachment Loss (CAL), Bleeding on Probing (BOP), Periodontal Inflamed Surface Area (PISA), periopathogens (P. gingivalis IgG/gingipains).	
Concept (C)	Domain 2 (Block 2):Nutritional, Metabolic & Systemic Biomarkers	• One-Carbon Metabolism: Vitamin B12, folate, total plasma homocysteine (tHcy).	Candidate Mediators & Systemic Intermediates: Laboratory, somatic, and metabolic parameters acting as potential biological intermediates connecting oral breakdown to cognitive outcomes.
		• Systemic Inflammation: C-Reactive Protein (CRP), Interleukin-6 (IL-6), TNF-α.	
		• Somatic & Protein Status: Serum albumin, Body Mass Index (BMI), undernutrition indices (MNA, GNRI), dietary intake quality (fiber, fruit/vegetable intake).	
Concept (C)	Domain 3 (Block 3):Neurological & Cognitive Endpoints	• Global Cognitive Screening: MMSE, MoCA, MCE-35.	Target Endpoints: Objective clinical, neuropsychological, and structural correlates of cognitive vulnerability and neurodegeneration.
		• Clinical Diagnostic Categories: Mild Cognitive Impairment (MCI), all-cause dementia, Alzheimer’s Disease (AD/ADRD).	
		• Domain-Specific Neuropsychology: Memory trajectories, executive function, attention.	
		• Neuroimaging & Fluid Biomarkers: Regional hippocampal atrophy, grey matter volume, white matter hyperintensities (WMH), PET amyloid-β/tau deposition.	
Context (C)	Setting & Geographical Scope	Any epidemiological, clinical, or community setting globally (community senior centers, long-term care residences, memory clinics, national administrative databases).	Inclusion Rule: Human clinical and observational evidence published in English between January 1, 2010, and June 30, 2025.
Study Design Filters	Eligible Evidence Types	Prospective cohort studies, cross-sectional studies, retrospective database cohorts, case-control studies, interventional trials/prosthetic RCTs.	Multi-Domain Threshold: Studies were required to evaluate at least two of the three core ONBA domains simultaneously. Narrative reviews, editorials, animal studies, and single-domain studies were excluded.

Note: Studies were required to examine Domain 1 in combination with Domain 2 and/or Domain 3. Abbreviations: AD = Alzheimer’s Disease; ADRD = Alzheimer’s Disease and Related Dementias; BOP = Bleeding on Probing; CAL = Clinical Attachment Loss; CRP = C-Reactive Protein; DMFT = Decayed, Missing, Filled Teeth; FMUs = Functional Masticatory Units; GNRI = Geriatric Nutritional Risk Index; IL-6 = Interleukin-6; MNA = Mini-Nutritional Assessment; MMSE = Mini-Mental State Examination; MoCA = Montreal Cognitive Assessment; ONBA = Oral–Nutrition–Brain Axis; PCC = Population, Concept, and Context; PISA = Periodontal Inflamed Surface Area; PPD = Probing Pocket Depth; tHcy = Total Plasma Homocysteine; TNF-α = Tumor Necrosis Factor-α.

**Table 2:** Overall methodological, geographic, and demographic characteristics (n = 120).

Category Domain	Subcategory/Classification	Studies (n)	Percentage (%)	Cumulative Sample (N)	Representative Cohorts/Examples
**Study Design**	Cross-Sectional Study	63	52.5	104,215	SONIC, Concord Health, Zabut
	Prospective Cohort Study	40	33.3	285,410	Hisayama, AGES, Ohasama, CLHLS
	Retrospective Cohort Study	6	5.0	142,800	Taiwanese NHIRD, Korean NHIS
	Case-Control Study	5	4.2	1850	Vancouver LTC, Fujiwara-kyo
	Systematic Review/Meta-Analysis	4	3.3	N/A	JBI/PRISMA Syntheses
	Clinical Trial/Interventional Study	2	1.7	149	Complete Denture RCTs
**Geographic Region**	East Asia (Japan, China, S. Korea, TW)	68	56.7	285,400	Hisayama, SONIC, CLHLS, LIFE
	Europe (Finland, UK, NL, IT, SE)	18	15.0	92,150	ELSA, Gothenburg, Zabut
	North America (USA, Canada)	13	10.8	148,200	Health ABC, HRS, HABS-HD
	Global/Multi-Region	10	8.3	5200	Systematic Reviews
	South Asia/Middle East	5	4.2	2850	UAE, India, Turkey
	South America (Brazil)	3	2.5	182	Recife/São Paulo Cohorts
	Oceania (Australia)	3	2.5	1700	Sydney Memory & Ageing, CHAMP
**Population Setting**	Community-dwelling older adults	90	75.0	485,320	Population checkups, JAGES
	Nursing home/Long-term care	18	15.0	32,150	Institutionalized elders
	Memory clinic/Outpatients	12	10.0	16,950	MCI & AD outpatient clinics
**Cognitive Assessment**	Clinical Diagnosis (MCI, AD, ADRD)	86	71.7	380,400	DSM-IV, NINCDS-ADRDA, ICD-10
	Global Screening (MMSE, MoCA)	67	55.8	295,100	MMSE, MoCA, MCE-35
	Neuropsychological Battery	32	26.7	120,400	WMS-III, Trail Making, Fluency
	Neuroimaging/Fluid Biomarkers	13	10.8	14,200	MRI Volume, PET Tau/Aβ, CSF

Abbreviations: AD = Alzheimer’s Disease; ADRD = Alzheimer’s Disease and Related Dementias; AGES = Aichi Gerontological Evaluation Study; CHAMP = Concord Health and Ageing in Men Project; CLHLS = Chinese Longitudinal Healthy Longevity Survey; ELSA = English Longitudinal Study of Ageing; HABS-HD = Health and Aging Brain Study–Health Disparities; MCI = Mild Cognitive Impairment; MMSE = Mini-Mental State Examination; MoCA = Montreal Cognitive Assessment.

**Table 3:** Summary of mapped evidence across oral indicators, nutritional biomarkers, and proposed ONBA axes (n = 120).

Domain Axis/Biomarker	Studies (n)	%	Common Statistical Role	Evidence Interpretation	Primary Mapped Cognitive Finding
**Anatomical Dentition**					
• Tooth Loss (<20 natural teeth)	94	78.3	Primary Exposure	Prospective & Cross-Sectional	Dose-response link with incident MCI/dementia.
• Complete Edentulism	28	23.3	Primary Exposure	Observational Association	Correlates with clinical dementia severity.
• DMFT Index / Dental Caries	12	10.0	Primary Exposure	Cross-Sectional Association	Active caries correlates with memory drop.
**Masticatory Function**					
• Subjective Chewing Disability	42	35.0	Primary Exposure	Longitudinal Association	Predicts 5-year incident cognitive decline.
• Maximal Bite / Occlusal Force	21	17.5	Functional Metric	Observational & Longitudinal	Lower bite force predicts 3-year MMSE decline.
• Chewing Efficiency (Mixing Tests)	18	15.0	Functional Metric	Cross-Sectional Association	Reduced mixing ability linked to lower MoCA.
• Functional Masticatory Units	15	12.5	Functional Metric	Candidate Mediator	Higher FMU count protective against impairment.
**Prosthetic Rehabilitation**					
• Complete/Partial Denture Wear	48	40.0	Moderator/Buffer	Longitudinal & Interventional	Denture wear attenuates tooth loss risk.
• Prosthetic Fit / Quality Index	12	10.0	Moderator/Buffer	Cross-Sectional Association	Unsatisfactory functional quality lowers MMSE.
**Periodontal Disease**					
• Severe Periodontitis (PPD/CAL)	88	73.3	Primary Exposure	Prospective Cohort Evidence	Severe attachment loss increases dementia risk.
• Bleeding on Probing (BOP)	35	29.2	Inflammatory Marker	Cross-Sectional Association	Local gingival inflammation tracks lower memory.
• Periodontal Inflamed Surface Area	14	11.7	Composite Risk Index	Cross-Sectional Association	PISA scores track advancing dementia stages.
• P. gingivalis Antibodies/Gingipains	18	15.0	Microbial Exposure	Serological & Pathological	Seropositivity linked to AD risk and tau deposition.
**Nutritional & Metabolic Biomarkers**					
• BMI / Low Body Weight / Frailty	42	35.0	Candidate Mediator	Path Analysis/Mediation	Low BMI mediates tooth loss-cognition link.
• Dietary Intake (Fiber/Fruits/Veg)	28	23.3	Candidate Mediator	Longitudinal Path Analysis	Soft-diet transition mediates B-vitamin drop.
• Serum Albumin / Malnutrition	18	15.0	Systemic Intermediate	Cross-Sectional Association	Hypoalbuminemia co-occurs with masticatory failure.
• Systemic Inflammation (CRP/IL-6)	18	15.0	Candidate Mediator	Multivariable Mediation	CRP/IL-6 mediate periodontal spillover.
• B-Vitamins (B12/Folate/Homocysteine)	8	6.70	Metabolic Intermediate	Observational & Metabolic	B12/folate drop and high tHcy link loss to atrophy.
**Proposed ONBA Axes (Conceptual)**					
• Sensorimotor Deafferentation Axis	111	92.5	Hypothesized Mechanism	Neuroimaging & Physiological	Occlusal loss reduces trigeminal feedback/CBF.
• Nutritional–Metabolic Axis	60	50.0	Hypothesized Mechanism	Metabolic & Path Analysis	Soft diet drives B-vitamin drop & hyperhomocysteinemia.
• Inflammatory–Microbial Axis	48	40.0	Hypothesized Mechanism	Inflammatory & Serological	Periodontitis drives systemic CRP & BBB penetration.

Abbreviations: BOP = Bleeding on Probing; CAL = Clinical Attachment Loss; CBF = Cerebral Blood Flow; DMFT = Decayed, Missing, Filled Teeth; FMUs = Functional Masticatory Units; IL-6 = Interleukin-6; PISA = Periodontal Inflamed Surface Area; PPD = Probing Pocket Depth; tHcy = Total Plasma Homocysteine.

## Data Availability

No new data were created or analyzed in this study. Data sharing is not applicable to this article.

## Supplementary Materials

The following supporting information can be downloaded at: https://www.mdpi.com/article/10.3390/nu18213637/s1, Table S1: The completed PRISMA-ScR checklist, Table S2: Search Strategy & Search Terms.
