# Supplementary material for: Mapping the Oral–Nutrition–Brain Axis in Aging: A Scoping Review of Evidence Linking Oral Health, Nutrition, and Cognitive Outcomes
Source: Nutrients. 2026 Oct 31;18(21):3637. doi: 10.3390/nu18213637 (PMC13764962; doi:10.3390/nu18213637)
Supplement: Supplementary file 1 [file nutrients-18-03637-s001.zip › nutrients-4561351-supplementary.pdf]

PRISMA

# PRISMA 2020 Checklist

## Supplementary Table S1: The completed PRISMA-ScR checklist

| Section and Topic | Item # | Checklist item | Location where item is reported |
|---|---|---|---|
| **TITLE** | | | |
| Title | 1 | Identify the report as a systematic review. | **Title:** *"Mapping the Oral–Nutrition–Brain Axis in Aging: A Scoping Review of Evidence Linking Oral Health, Nutrition, and Cognitive Outcomes"* |
| **ABSTRACT** | | | |
| Abstract | 2 | See the PRISMA 2020 for Abstracts checklist. | **Abstract:** Structured into Background, Objective, Methods, Results, and Conclusions. |
| **INTRODUCTION** | | | |
| Rationale | 3 | Describe the rationale for the review in the context of existing knowledge. | **Introduction**Paragraphs 1–4 detail the global burden of oral diseases/dementias and the critical gap in traditional neurodegenerative frameworks that omit upstream oral/nutritional markers. |
| Objectives | 4 | Provide an explicit statement of the objective(s) or question(s) the review addresses. | **Section 1 (Introduction):** *"By moving beyond traditional central neurological markers alone, this scoping review (ScR) aims to systematically map, synthesize, and identify evidence gaps regarding how oral health factors, nutritional biomarkers, and cognitive outcomes interact in older adults (≥65 years)..."* |
| **METHODS** | | | |
| Eligibility criteria | 5 | Specify the inclusion and exclusion criteria for the review and how studies were grouped for the syntheses. | **Section 2.1 & 2.2 —** *PCC Framework (Population aged ≥65, Concept covering ONBA Blocks 1–3 requiring at least two domains, Context).* |
| Information sources | 6 | Specify all databases, registers, websites, organisations, reference lists and other sources searched or consulted to identify studies. Specify the date when each source was last searched or consulted. | **Section 2.3 —** *MEDLINE ALL, Embase, Web of Science Core Collection, Cochrane Database of Systematic Reviews, and Cochrane CENTRAL searched on June 17, 2025.* |
| Search strategy | 7 | Present the full search strategies for all databases, registers and websites, including any filters and limits used. | **Section 2.3 & Supplementary Table S2 (Table S2).** |
| Selection process | 8 | Specify the methods used to decide whether a study met the inclusion criteria of the review, including how many reviewers screened each record and each report retrieved, whether they worked independently, and if applicable, details of automation tools used in the process. | **Section 2.4 —** *EndNote 2025, Covidence, pilot testing on 20 citations, duplicate screening by* |

| Section and Topic | Item # | Checklist item | Location where item is reported |
|---|---|---|---|
| | | | *independent reviewers (HN, SD), and arbitration by senior reviewers (RK, SC).* |
| Data collection process | 9 | Specify the methods used to collect data from reports, including how many reviewers collected data from each report, whether they worked independently, any processes for obtaining or confirming data from study investigators, and if applicable, details of automation tools used in the process. | **Section 2.5 —** *Standardized Excel charting form piloted on 3 studies, duplicate extraction (HN, SD) with consensus verification (SC).* |
| Data items | 10a | List and define all outcomes for which data were sought. Specify whether all results that were compatible with each outcome domain in each study were sought (e.g. for all measures, time points, analyses), and if not, the methods used to decide which results to collect. | **Table 1:** Target Endpoints (Block 3) defining MMSE, MoCA, clinical diagnostic categories (MCI, AD, ADRD), domain batteries, and PET/MRI neuroimaging markers. |
| | 10b | List and define all other variables for which data were sought (e.g. participant and intervention characteristics, funding sources). Describe any assumptions made about any missing or unclear information. | **Table 1:** Primary Exposures (Block 1: natural tooth count, edentulism, DMFT, bite force, chewing efficiency, FMUs, prostheses, PPD, CAL, BOP, PISA) and Systemic Mediators (Block 2: B12, folate, homocysteine, albumin, CRP, IL-6, TNF-$\alpha$, BMI, MNA, GNRI). |
| Study risk of bias assessment | 11 | Specify the methods used to assess risk of bias in the included studies, including details of the tool(s) used, how many reviewers assessed each study and whether they worked independently, and if applicable, details of automation tools used in the process. | **N/A (Scoping Review / JBI Framework): Section 3.1** explicitly states: *"In accordance with updated JBI guidance and PRISMA-ScR standards, formal critical appraisal or risk of bias assessment was not performed, as the primary objective of this scoping review was to map and characterize the available evidence landscape..."* |
| Effect measures | 12 | Specify for each outcome the effect measure(s) (e.g. risk ratio, mean difference) used in the synthesis or presentation of results. | **N/A (Scoping Review / JBI Framework):** Formal effect size pooling was not performed. Descriptive frequency counts ($n$, %), cumulative sample sizes ($N$), and qualitative directional mapping are presented in **Tables 2, 3, and 4**. |
| Synthesis methods | 13a | Describe the processes used to decide which studies were eligible for each synthesis (e.g. tabulating the study intervention characteristics and comparing against the planned groups for each synthesis (item #5)). | **Table 1:** Categorization based on meeting PCC criteria across the three biological domains of the ONBA. |
| | 13b | Describe any methods required to prepare the data for presentation or synthesis, such as handling of missing summary statistics, or data conversions. | Standardization and harmonization of oral exposures, biomarker assays, and cognitive screening categories into 3 unified block |

| Section and Topic | Item # | Checklist item | Location where item is reported |
|---|---|---|---|
| | 13c | Describe any methods used to tabulate or visually display results of individual studies and syntheses. | Visually presented via **Figure 1** (PRISMA Flowchart), **Figure 2** (Conceptual Pathway Framework), **Table 1**(PICO Concepts), **Tables 2 & 3** (Methodological/Geographic Characteristics), **Table 4** (Domain Summary), and **Table S2** (Biomarker Fingerprints). |
| | 13d | Describe any methods used to synthesize results and provide a rationale for the choice(s). If meta-analysis was performed, describe the model(s), method(s) to identify the presence and extent of statistical heterogeneity, and software package(s) used. | Qualitative conceptual mapping and descriptive statistics (frequency counts and percentages) organized around the three predefined biological domains. |
| | 13e | Describe any methods used to explore possible causes of heterogeneity among study results (e.g. subgroup analysis, meta-regression). | Methodological breakdowns by study design, geographical distribution (East Asia vs. Western nations), population setting (community vs. nursing home/memory clinic), and cognitive metric type. |
| | 13f | Describe any sensitivity analyses conducted to assess robustness of the synthesized results. | **N/A (Scoping Review / JBI Framework):** Quantitative sensitivity analysis is not applicable for qualitative scoping reviews. |
| Reporting bias assessment | 14 | Describe any methods used to assess risk of bias due to missing results in a synthesis (arising from reporting biases). | **N/A (Scoping Review / JBI Framework):** Publication bias assessment is omitted by design per JBI scoping review guidelines. |
| Certainty assessment | 15 | Describe any methods used to assess certainty (or confidence) in the body of evidence for an outcome. | **N/A (Scoping Review / JBI Framework):** GRADE certainty assessment is omitted by design per PRISMA-ScR standards. |
| **RESULTS** | | | |
| Study selection | 16a | Describe the results of the search and selection process, from the number of records identified in the search to the number of studies included in the review, ideally using a flow diagram. | **Section 3.1 & Figure 1 —** *3,415 records identified; 1,988 unique records screened; 763 full-text articles assessed; 120 studies included*. |
| | 16b | Cite studies that might appear to meet the inclusion criteria, but which were excluded, and explain why they were excluded. | **Section 3.1 & Supplementary Materials:** Specific exclusion counts (wrong age $n = 216$, wrong outcomes $n = 71$, wrong study design $n = 68$, etc.) listed in **Figure 1** and documented in **Supplementary Table S1**. |

| Section and Topic | Item # | Checklist item | Location where item is reported |
|---|---|---|---|
| Study characteristics | 17 | Cite each included study and present its characteristics. | All 120 included studies are cited and summarized across methodological, geographic, exposure, biomarker, and pathway categories. |
| Risk of bias in studies | 18 | Present assessments of risk of bias for each included study. | **N/A (Scoping Review / JBI Framework):** Omitted by design per JBI scoping review methodology. |
| Results of individual studies | 19 | For all outcomes, present, for each study: (a) summary statistics for each group (where appropriate) and (b) an effect estimate and its precision (e.g. confidence/credible interval), ideally using structured tables or plots. | **Section 3.2–3.4 & Table 3 —** *Direction of effect, evidence interpretations, and associated reference groupings*. |
| Results of syntheses | 20a | For each synthesis, briefly summarise the characteristics and risk of bias among contributing studies. | **Section 3.1 —** *Summarizes that 94.2% were observational, 56.7% conducted in East Asia, 75.0% evaluated community-dwelling older adults, and 71.7% utilized clinical AD/ADRD diagnostic classifications*. |
| | 20b | Present results of all statistical syntheses conducted. If meta-analysis was done, present for each the summary estimate and its precision (e.g. confidence/credible interval) and measures of statistical heterogeneity. If comparing groups, describe the direction of the effect. | **N/A (Scoping Review / JBI Framework):** Qualitative scoping review; summary of primary cognitive findings and directional effects mapped in **Table 4**. |
| | 20c | Present results of all investigations of possible causes of heterogeneity among study results. | **Section 4.1 & 4.3 —** *Contrasts across geographical regions (East Asia vs. Western cohorts) and healthcare structures (universal coverage vs. US Medicare dental exclusions)*. |
| | 20d | Present results of all sensitivity analyses conducted to assess the robustness of the synthesized results. | N/A (Scoping Review / JBI Framework) |
| Reporting biases | 21 | Present assessments of risk of bias due to missing results (arising from reporting biases) for each synthesis assessed. | N/A (Scoping Review / JBI Framework) |
| Certainty of evidence | 22 | Present assessments of certainty (or confidence) in the body of evidence for each outcome assessed. | N/A (Scoping Review / JBI Framework) |
| **DISCUSSION** | | | |
| Discussion | 23a | Provide a general interpretation of the results in the context of other evidence. | **Section 4 (Discussion) —** *Synthesis of evidence supporting the ONBA across the 3 conceptual pathways (Inflammatory-Microbial, Nutritional-Metabolic, and Sensorimotor Deafferentation)*. |

| Section and Topic | Item # | Checklist item | Location where item is reported |
|---|---|---|---|
| | 23b | Discuss any limitations of the evidence included in the review. | **Section 4.4 (Methodological Limitations) —** Explicitly details language restriction bias, observational dominance (94.2%), absence of critical appraisal per JBI scoping standards, cumulative sample tallying caution, exposure/outcome heterogeneity, a priori framework influence, domain multiplicity threshold, and conceptual mediation vs. causal testing. |
| | 23c | Discuss any limitations of the review processes used. | |
| | 23d | Discuss implications of the results for practice, policy, and future research. | **Section 4.3 & Section 4.5 (Future Perspectives)** *— Reorganization of geriatric clinical protocols, expansion of public dental coverage, integration of oral screening in memory clinics, and operationalization in longitudinal multi-ethnic cohorts like HABS-HD.* |
| **OTHER INFORMATION** | | | |
| Registration and protocol | 24a | Provide registration information for the review, including register name and registration number, or state that the review was not registered. | **Section 2.1 —** *Conducted according to an a priori prospective protocol publicly registered on the Open Science Framework (OSF) repository.* |
| | 24b | Indicate where the review protocol can be accessed, or state that a protocol was not prepared. | |
| | 24c | Describe and explain any amendments to information provided at registration or in the protocol. | |
| Support | 25 | Describe sources of financial or non-financial support for the review, and the role of the funders or sponsors in the review. | **Funding Section —** *Supported by HESP University of Wisconsin-Madison and NIH/NIA Award Numbers R01AG054073, R01AG058533, P41EB015922, and U19AG078109 (HABS-HD).* |
| Competing interests | 26 | Declare any competing interests of review authors. | Conflicts of Interest Section— *Declared as none.* |
| Availability of data, code and other materials | 27 | Report which of the following are publicly available and where they can be found: template data collection forms; data extracted from included studies; data used for all analyses; analytic code; any other materials used in the review. | **Supplementary Materials Section —** *Documents search strategies (Table S1/S2) and biomarker tables (Table S3).* |

*From:* Page MJ, McKenzie JE, Bossuyt PM, Boutron I, Hoffmann TC, Mulrow CD, et al. The PRISMA 2020 statement: an updated guideline for reporting systematic reviews. BMJ 2021;372:n71. doi: 10.1136/bmj.n71. This work is licensed under CC BY 4.0. To view a copy of this license, visit https://creativecommons.org/licenses/by/4.0/

| | |
|---|---|
| **Supplementary Table S2. Search Strategy & Search Terms**<br>**Topic:** The impact of oral health on dementia in older adults<br>**Team:** Dr. Steffany Chamut<br>**Search developed by:** Ellie Svoboda, MLIS<br>**Review type:** Tier 1<br>**Databases:** MEDLINE (Ovid); Embase; Web of Science; Cochrane CENTRAL; Cochrane Library<br>**Search date:** 6/17/2025<br>**Total references:** 3415<br>**Total following deduplication:** 1993<br>**Software:** Endnote; Covidence | |
| Ovid Medline | 1. ((nutrition* or biomarker* or marker*) adj8 (vitamin b* or B12 or folate* or folic acid* or vitamin m or Cyanocobalamin or Cobalamin)).tw,kf.<br>2. (exp "Nutritional Status"/ or exp Biomarkers/) and (exp "Vitamin B Complex"/ or exp "Vitamin B 12"/ or exp "Folic Acid"/)<br>3. 1 or 2<br>4. (((tooth or teeth) adj4 (count* or loss* or function* or number* or missing)) or dentition* or masticat* or chew*).tw,kf.<br>5. exp "Tooth Loss"/ or exp Mastication/<br>6. 4 or 5<br>7. 3 or 6<br>8. (cognit* or dementia* or alzheimer* or alzeimer* or aphasia* or lewy bod*).tw,kf.<br>9. exp Dementia/ or exp "Cognition Disorders"/<br>10. 8 or 9<br>11. (aging or ageing or elder* or (older adj2 adult*) or geriatr* or gerontolog* or senior* or senescen* or retire* or sexagenarian* or septuagenarian* or octogenarian* or nonagenarian* or centenarian* or supercentenarian* or Medicare or advanced age).tw,kf.<br>12. aging/ or exp aged/ or geriatrics/<br>13. 11 or 12<br>14. 7 and 10 and 13<br>15. limit 14 to english language<br>16. limit 15 to yr="2010 -Current" |
| Embase | 1. ((nutrition* OR biomarker* OR marker*) NEAR/8 ('vitamin b*' OR b12 OR folate* OR 'folic acid*' OR 'vitamin m' OR cyanocobalamin OR cobalamin)):ti,ab,kw<br>2. ('nutritional status'/exp OR 'biological marker'/exp) AND 'vitamin b group'/exp<br>3. #1 OR #2<br>4.(((tooth OR teeth) NEAR/4 (count* OR loss* OR function* OR |

| | |
|---|---|
| | number* OR missing)):ti,ab,kw) OR dentition*:ti,ab,kw OR masticat*:ti,ab,kw OR chew*:ti,ab,kw<br>5. 'periodontal disease'/exp OR 'mastication'/exp<br>6. #4 OR #5<br>7. #3 OR #6<br>8. cognit*:ti,ab,kw OR dementia*:ti,ab,kw OR alzheimer*:ti,ab,kw OR alzeimer*:ti,ab,kw OR aphasia*:ti,ab,kw OR 'lewy bod*':ti,ab,kw<br>9. 'dementia'/exp OR 'cognitive defect'/exp<br>10. #8 OR #9<br>11. aging:ti,ab,kw OR ageing:ti,ab,kw OR elder*:ti,ab,kw OR ((older NEAR/2 adult*):ti,ab,kw) OR geriatr*:ti,ab,kw OR gerontolog*:ti,ab,kw OR senior*:ti,ab,kw OR senescen*:ti,ab,kw OR retire*:ti,ab,kw OR sexagenarian*:ti,ab,kw OR septuagenarian*:ti,ab,kw OR octogenarian*:ti,ab,kw OR nonagenarian*:ti,ab,kw OR centenarian*:ti,ab,kw OR supercentenarian*:ti,ab,kw OR medicare:ti,ab,kw OR 'advanced age':ti,ab,kw<br>12. 'aging'/exp OR 'aged'/exp OR 'geriatrics'/exp<br>13. #11 OR #12<br>14. #7 AND #10 AND #13<br>15. #7 AND #10 AND #13 AND [english]/lim<br>16. #7 AND #10 AND #13 AND [english]/lim AND [2010-2025]/py<br>17. #7 AND #10 AND #13 AND [english]/lim AND [2010-2025]/py AND ('conference abstract'/it OR 'conference paper'/it OR 'conference review'/it OR 'editorial'/it OR 'letter'/it)<br>18. #16 NOT #17 |
| Web of Science | 1. TS=((nutrition* OR biomarker* OR marker*) NEAR/8 ("vitamin b*" OR B12 OR folate* OR "folic acid*" OR "vitamin m" OR Cyanocobalamin OR Cobalamin))<br>2. TS=(((tooth OR teeth) NEAR/4 (count* OR loss* OR function* OR number* OR missing)) OR dentition* OR masticat* OR chew*)<br>3. TS=(cognit* OR dementia* OR alzheimer* OR alzeimer* OR aphasia* OR "lewy bod*")<br>4. TS=(aging OR ageing OR elder* OR (older NEAR/2 adult*) OR geriatr* OR gerontolog* OR senior* OR senescen* OR retire* OR sexagenarian* OR septuagenarian* OR octogenarian* OR nonagenarian* OR centenarian* OR supercentenarian* OR Medicare OR "advanced age")<br>5. #1 OR #2<br>6. #3 AND #4 AND #5<br>7. #3 AND #4 AND #5 and English (Languages)<br>8. #3 AND #4 AND #5 and English (Languages) and 2025 or 2024 or 2023 or 2022 or 2021 or 2020 or 2019 or 2018 or 2017 or 2016 or 2015 or 2014 or 2013 or 2012 or 2011 or 2010 (Publication Years) |

| | |
|---|---|
| Cochrane Library and Cochrane CENTRAL | 1. ((nutrition*:ti,ab,kw OR biomarker*:ti,ab,kw OR marker*:ti,ab,kw) NEAR/8 (("vitamin" NEXT b*):ti,ab,kw OR B12:ti,ab,kw OR folate*:ti,ab,kw OR ("folic" NEXT acid*):ti,ab,kw OR "vitamin m":ti,ab,kw OR Cyanocobalamin:ti,ab,kw OR Cobalamin:ti,ab,kw))<br>2. MeSH descriptor: [Nutritional Status] explode all trees<br>3. MeSH descriptor: [Biomarkers] explode all trees<br>4. #2 OR #3<br>5. MeSH descriptor: [Vitamin B Complex] explode all trees<br>6. MeSH descriptor: [Vitamin B 12] explode all trees<br>7. MeSH descriptor: [Folic Acid] explode all trees<br>8. #5 OR #6 OR #7<br>9. #4 AND #8<br>10. #1 OR #9<br>11. (((tooth:ti,ab,kw OR teeth:ti,ab,kw) NEAR/4 (count*:ti,ab,kw OR loss*:ti,ab,kw OR function*:ti,ab,kw OR number*:ti,ab,kw OR missing:ti,ab,kw)) OR dentition*:ti,ab,kw OR masticat*:ti,ab,kw OR chew*:ti,ab,kw)<br>12. MeSH descriptor: [Tooth Loss] explode all trees<br>13. MeSH descriptor: [Mastication] explode all trees<br>14. #11 OR #12 OR #13<br>15. #10 OR #14<br>16. (cognit*:ti,ab,kw OR dementia*:ti,ab,kw OR alzheimer*:ti,ab,kw OR alzeimer*:ti,ab,kw OR aphasia*:ti,ab,kw OR ("lewy" NEXT bod*):ti,ab,kw)<br>17. MeSH descriptor: [Dementia] explode all trees<br>18. MeSH descriptor: [Cognition Disorders] explode all trees<br>19. #16 OR #17 OR #18<br>20. (aging:ti,ab,kw OR ageing:ti,ab,kw OR elder*:ti,ab,kw OR (older:ti,ab,kw NEAR/2 adult*:ti,ab,kw) OR geriatr*:ti,ab,kw OR gerontolog*:ti,ab,kw OR senior*:ti,ab,kw OR senescen*:ti,ab,kw OR retire*:ti,ab,kw OR sexagenarian*:ti,ab,kw OR septuagenarian*:ti,ab,kw OR octogenarian*:ti,ab,kw OR nonagenarian*:ti,ab,kw OR centenarian*:ti,ab,kw OR supercentenarian*:ti,ab,kw OR Medicare:ti,ab,kw OR "advanced age":ti,ab,kw)<br>21. MeSH descriptor: [Aged] explode all trees<br>22. MeSH descriptor: [Aged] explode all trees<br>23. MeSH descriptor: [Geriatrics] explode all trees<br>24. #20 OR #21 OR #22 OR #23<br>25. #15 AND #19 AND #24 within Cochrane Library publication date Between Jan 2010 and Jun 2025 |
